# Prediabetes and type 2 diabetes but not obesity are associated with alterations in bile acid related gut microbe-microbe and gut microbe-host community metabolism

**DOI:** 10.1080/19490976.2025.2474143

**Published:** 2025-03-05

**Authors:** Kristina Schlicht, Lea Pape, Nathalie Rohmann, Carina Knappe, Johannes Epe, Corinna Geisler, Daniela Pohlschneider, Susanne Brodesser, Lucy Kruse, Maria-Elisabeth Rohlfing, Katharina Hartmann, Kathrin Türk, Jens Marquardt, Jan Beckmann, Witigo von Schönfels, Alexia Beckmann, Perdita Wietzke-Braun, Dominik M. Schulte, Tim Hollstein, Tobias Demetrowitsch, Julia Jensen-Kroll, Fynn Brix, Stefan Schreiber, Andre Franke, Karin Schwarz, Silvio Waschina, Matthias Laudes

**Affiliations:** a Institute of Diabetes and Clinical Metabolic Research, University Medical Center Schleswig-Holstein, Kiel, Germany; b Faculty of Medicine and University Hospital of Cologne, Cluster of Excellence Cellular Stress Responses in Aging-associated Diseases (CECAD), University of Cologne, Cologne, Germany; c Department of Internal Medicine 1, University Medical Center Schleswig-Holstein, Lübeck, Germany; d Department of General and Abdominal Surgery, University Medical Center Schleswig-Holstein (UKSH), Kiel, Germany; e Division of Endocrinology, Diabetes and Clinical Nutrition, Department of Internal Medicine I, University Medical Center Schleswig-Holstein, Kiel, Germany; f Division of Food Technology, Institute of Human Nutrition and Food Science, Kiel University, Kiel, Germany; g Institute of Clinical Molecular Biology, Kiel University, Kiel, Germany

**Keywords:** Bile acids, type 2 diabetes, gut microbiome, gut community metabolism

## Abstract

The interplay between bile acids (BAs) and metabolic diseases has gained importance in recent years, with a variety of studies investigating their relationship with diverging results. Therefore, in the present study we performed a detailed analysis of BA metabolism in 492 subjects with different metabolic phenotypes. Besides microbiomics and metabolomics this investigation included *in silico* analysis of community metabolism to examine metabolic interchange between different microbes as well as microbes and the human host. Our findings revealed distinct changes in the BA profiles of patients with diabetes and prediabetes, whereas obesity alone had no influence on circulating BAs. Impaired glycemic control led to increased circulating BAs, a shift toward more secondary BAs, and an increase in the ratio of glycine to taurine-conjugated BAs. Additional analyses revealed that the ratio of glycine to taurine conjugation demonstrated variations between the single BAs, cholic acid (CA), chenodeoxycholic acid (CDCA) and deoxycholic acid (DCA), regardless of the metabolic status, with CA having a higher fraction of taurine conjugation. Furthermore, we found that microbiome alterations are associated with BAs, independent of diabetes or obesity. Analysis of microbial community metabolism revealed differential relative pathway abundance in relation to diabetes, particularly those related to membrane and polyamine synthesis. Increased bacterial cross-feeding of polyamines, galactose, and D-arabinose also coincided with an increase in BA. Notably, our serum metabolome analysis mirrored several of the previously *in silico* predicted exchanged metabolites, especially amino acid metabolism. Therefore, targeting BA metabolism may be a future approach for the treatment of metabolic diseases, especially prediabetes and type 2 diabetes.

## Introduction

Bile acids (BAs) have been recognized as key endocrine signaling molecules in recent years. They are involved in the regulation of glucose, lipid, and energy homeostasis as well as inflammation.^1^ The primary BA receptors, farnesoid X receptor (FXR) and Takeda G protein-coupled receptor 5 (TGR5), have unique roles and expression patterns. FXR, present in various tissues including enterocytes, hepatocytes, and immune cells, regulates protein expression for BA synthesis, energy, lipid homeostasis, and immunity, and affects glucose metabolism and insulin sensitivity.^2–4^ TGR5, unlike FXR is expressed in the gastrointestinal tract in sinusoidal, Kupffer, and biliary epithelial cells but not in hepatocytes.^5^ Here, it exhibits anti-inflammatory properties and depresses interleukin-6 (IL-6) and tumor necrosis factor α (TNF-α) production.^6^ Activation of TGR5 in enteroendocrine L-cells in the intestine results in the secretion of glucagon-like peptide 1 (GLP1) that stimulates insulin secretion, promotes insulin sensitization in brown adipose tissue and muscle cells, and inhibits glucagon secretion.

The gut microbiome, a complex and dynamic system, is influenced by host age, lifestyle, diet, diseases, and pharmaceuticals. Alterations in gut microbiome composition are linked to numerous diseases, including obesity and diabetes.^7^ Diet is the most significant factor affecting gut microbiome composition; however, BAs also play a crucial role in shaping gut microbial communities. As potent detergents, BAs disrupt cell membranes, leading to cell death and exhibiting antimicrobial effects that support the intestinal innate immune system.^8^ Specifically, deoxycholic acid (DCA) has been noted for its ability to compromise bacterial integrity and apply selective pressure.^9,10^ Enterohepatic circulation allows microbially modified BAs to impact systemic circulation, with TGR5 being highly responsive to secondary BAs produced solely by gut microbes, illustrating host-microbiome interaction. Beyond their direct antimicrobial action, BAs enhance intestinal barrier and immune functions by regulating antimicrobial peptides and proteins (AMPP). FXR activation in the terminal ileum prevents bacterial overgrowth by promoting AMPP production.^8^

Current evidence regarding the role of BA in obesity is conflicting. Some studies show altered BA profiles in obese patients, characterized by increased synthesis and higher fasting blood concentrations of BAs,^11–13^ while others report no significant difference in fasting BA levels between obese and lean individuals.^14–16^ These discrepancies may stem from varied study designs and the inconsistent inclusion of patients with diabetes or prediabetes. Similarly, research on type 2 diabetes (T2D) reveals mixed findings on BA composition or concentration changes. While several studies suggest that diabetic patients have distinct BA profiles with higher serum concentrations of single or total BAs than healthy controls,^17–21^ other studies report no significant differences in total BA serum concentrations between the two groups.^16,22^ The comparability of these studies is limited by differences in the inclusion criteria for T2D and obese patients and the generally small sample sizes of the cohort studies.

The objective of this study is to provide a detailed examination of how obesity, prediabetes, and T2D affect fasting serum BA concentrations and composition. This analysis was performed using a subset of the extensive Food Chain Plus (FoCus) cohort, which is characterized by comprehensive patient data across various -omics layers, including untargeted metabolomics and microbiome analysis. Additionally, we modeled bacterial community metabolism to more thoroughly explore the impact of BAs on gut microbes. We then validated these models using metabolome data, aiming to delineate a more complete understanding of the interactions between BAs and the microbiome in both healthy and metabolic disease states.

## Methods

### Cohorts

The primary study population was sourced from the FoCus cohort,^23^ recruited between 2011 and 2015 at the Outpatient Center of the Division of Endocrinology, Diabetes and Clinical Nutrition of the University Medical Center Schleswig – Holstein (UKSH) Kiel and the regional registration office. This cohort provides comprehensive -omics data (phenomics, microbiomics, metabolomics, and genomics) from 2,000 participants. For this study, individuals taking cholekinetic or choleretic medications were excluded. All participants provided written informed consent.

Supplementary table S1: A summarizes bile acid distribution and characteristics within the FoCus subset. A total of 492 participants were included in this study, comprising 338 women and 154 men, with a median age of 53 years and with a BMI range from 14.33 to 70.52 (median 31.70). The study population was divided into four groups based on BMI and presence or absence of T2D: 19 participants in the underweight (UW) group (BMI <18.5 kg/m^2^), 191 in the normal weight (NW) group (BMI 18.5–24.9 kg/m^2^), 145 in the obese (OB) group (BMI >30 kg/m^2^, no T2D)), and 137 participants in the obese and diabetic group (OB_T2D) (BMI >30 kg/m^2^, T2D). Additionally, participants were categorized into three groups based on their medical history of T2D, independently of weight: (1) the healthy group, comprising 237 individuals with no T2D history and fasting blood glucose levels below 100 mg/dL; (2) the prediabetic group, with 112 participants having fasting blood glucose levels between 100 and 125 mg/dL; and (3) the diabetes group, consisting of 141 patients with a T2D history or fasting serum glucose levels >125 mg/dL. Group characteristics are presented in supplementary table S1:B.

In a secondary longitudinal cohort, we measured nine BAs in 46 patients who underwent bariatric surgery with measurements taken at baseline and six months post-intervention (EoI), and in 30 patients who completed a structured 6-month weight-loss program on a formula based very low-calorie diet. For the non-surgical group BA measurements were taken at baseline (BL), end of fasting (EoF, 3 months) and end of intervention (EoI, 6 months). Both intervention cohorts have been described in detail before.^24^

All probands provided written informed consent. Studies were approved by the Ethics committee of Kiel University.

### Bile acid measurement and imputation

Venous blood samples were analyzed to measure 9 BAs: cholic acid (CA), chenodeoxycholic acid (CDCA), deoxycholic acid (DCA), glycocholate (GCA), glycochenodeoxycholate (GCDCA), glycodeoxycholate (GDCA), taurocholate (TCA), taurochenodeoxycholate (TCDCA) and taurodeoxycholate (TDCA). These measurements were conducted using liquid chromatography/mass spectrometry (LC-MS) in a specialized laboratory (Medizinisches Labor Bremen, Bremen, Germany). In the intervention study BAs were quantified in the CECAD Lipidomics/Metabolomics Facility, University of Cologne, by Liquid Chromatography coupled to Electrospray Ionization Tandem Mass Spectrometry (LC-ESI-MS/MS) using the Biocrates® Bile Acids Kit (BIOCRATES Life Sciences AG, Innsbruck, Austria). In the FoCus cohort, overall 14.11% of observations had missing values, which were imputed using a K-nearest neighbor approach in the ‘Amelia’ package in R.^25^ Imputation was performed twice to handle values below the detection limit of 0.02 µM, ensuring imputed values remained within realistic ranges. The quality of imputation is evaluated in Supplement S2.

### Microbiomics

The stool samples were stored at − 80°C until analysis, which was conducted by the Institute for Clinical Molecular Biology (IKMB) in Kiel. Bacterial 16S ribosomal ribonucleic acid (rRNA) analysis was performed as described by Heinsen et al.^26^ First, 200 mg of stool was placed in bead-beating tubes (Garnet, 0.7 mm) filled with ASL lysis buffer and then homogenized using SpeedMill PLUS (Analytik Jena) for 45 s at 50 hz. The homogenized samples were then heated to 95°C for a duration of five minutes. Further preparations were performed according to the manufacturer’s instructions. The kits used for these procedures were the QIAamp DNA Stool Mini Kit and QIAcube system (Qiagen). Polymerase chain reaction (PCR) for amplification of the 16S rRNA gene was conducted using a pair of primers for the V1-V2 region, followed by normalization of deoxyribonucleic acid (DNA) concentrations using the SequalPrep Normalization Plate Kit (Thermo Fisher Scientific) according to the manufacturer’s protocol. Samples were pooled and sequenced using an Illumina MiSeq device. For more detailed information on this method see Geisler et al.^23^

### Metabolomics

Metabolomic analyses were conducted on both blood and urine samples. Urine samples were diluted 1:4 with water containing 0.1% formic acid before analysis with a quadrupole time-of-flight mass spectrometer (Bruker, Bremen, Germany). Metabolites from blood samples were extracted using a modified SIMPLEX approach, as described by Matyash et al.,^27^ and then analyzed with ultra-high-resolution Fourier Transform ion cyclotron resonance mass spectrometry (FT-ICR-MS, Bruker, Bremen, Germany). The semi-targeted evaluation utilized a local database based on the Kyoto Encyclopedia of Genes and Genomes (KEGG),^28^ HMDB,^29^ and relevant literature. To enhance reproducibility, metabolites present in less than 10% of samples or with counts below 1 million (for urine/blood) were excluded.

Mass spectrometry metabolomic analysis provides insights into a variety of metabolites. Rather than focusing on individual metabolites, we mapped the identified metabolites to metabolic pathways using the KEGG database, which offers a comprehensive collection of pathways linked to specific enzymes and metabolites across organisms. We conducted enrichment analysis to assess whether certain metabolic pathways were overrepresented among differentially expressed metabolites, comparing the observed number of metabolites in a pathway to the expected number by chance. This approach allows us to identify key pathways involved in the observed physiological or pathological conditions.

### Gut community metabolism

Metabolic pathways and genome-scale metabolic network models were predicted for all microbial genomes from the Human Reference Gut Microbiome (HRGM) resource using gapseq^30^ version 1.2 as described previously.^31^ To estimate pathway abundance in individual microbiomes, we summed the relative abundance of organisms within the HRGM collection that were predicted to have a respective pathway. Therefore, inferred amplicon sequence variants (ASVs) were mapped to the 16S ribosomal RNA genes within the genomes of the HRGM genome collection. Mapping was performed based on pairwise sequence alignments using BLASTN (version 2.9.0+)^32^ with a minimum query (ASV) coverage of 95% and a minimum sequence identity of 97%. In the case of multiple hits, only hits with the maximum identity were retained for further analysis.

To predict microbial community metabolic processes (i.e., ‘fluxes’), the individual metabolic network models of bacteria found with a relative abundance of > 0.5% in the respective sample were merged into multispecies models as described previously.^33–35^ In brief, the metabolic models for each species were linked through a common extracellular compartment, which enables the potential exchange of metabolites between organisms. The metabolic reactions of each species were represented in a separate compartment. This approach allows the preservation of species-specific metabolic capabilities and thereby captures the diversity of metabolic functions across different microbial species. Based on the multispecies models, metabolic fluxes, including rates of metabolite production, metabolite consumption, and metabolite cross-feeding, were predicted using parsimonious flux balance analysis (pFBA).^36^ Before flux predictions, additional ‘coupling constraints’ were added to the multispecies metabolic model as developed by Heinken et al.^37^ These coupling constraints aim to prevent predictions where reactions of one species carry a high flux only to cross-feed the product metabolites to other community members without contributing to the species’ own biomass formation. As the objective function for the pFBA, we defined the maximization of the summed biomass reaction fluxes of all individual species minus the total sum of absolute reaction fluxes scaled by a factor of 10^−6^. The summed (community) biomass reaction was defined to recapitulate also the relative abundances of species by setting fixed relative contributions (*b*_*i*_) by each species (*i*) to the overall community biomass. The relative contributions (*b*_*i*_) were set to the observed relative abundance of the species in the respective sample to account for the community composition in flux predictions. Predicted rates of metabolite production, consumption, and cross-feeding were normalized by dividing flux rates by the predicted community growth rate.

## Results

### Bile acid concentration in obesity, prediabetes and type 2 diabetes

**Figure 1.**
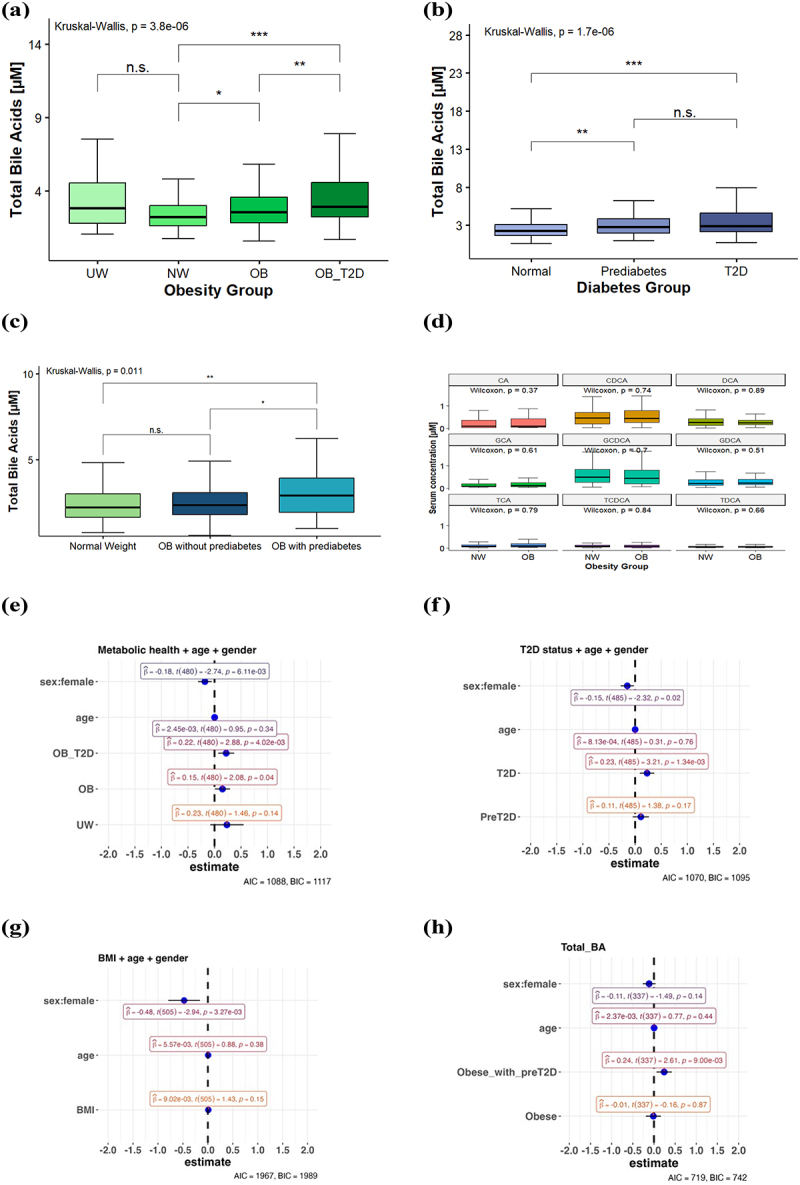
Total bile acid concentration in the FoCus cohort (a) Comparison of underweight, normal weight and obese patients (b) Comparison of prediabetic, diabetic and healthy participants. c) Total bile acid concentration in normal weight, obese patients with and without prediabetes (d) Comparison of single bile acids in normal weight and obese patients without pre/diabetes. (e-h) Generalized linear regression evaluating the impact of gender, age and (e)metabolic health groups/f)T2D status/g)bmi/h)obesity with and without preT2D on total bile acids as the dependent variable.

In the literature, the total BA serum concentration in healthy individuals is approximately 2.5 µmol/L.^38^ In our subgroup of normal weight participants, the average bile acid concentration was 2.88 µmol/L. For the entire study population, which included normal weight and obese subjects, the mean circulating BAs rose to 3.31 µmol/L. An upward trend in total BA concentration was observed across obesity groups, as shown in Figure 1(a). Patients with diabetes in our cohort also exhibited higher serum BA levels than the normal-weight control group, though there were no significant differences between patients with prediabetes and diabetes (Figure 1(b)). We also compared obese patients with normal glycemic control with the metabolically healthy NW group. When diabetic and prediabetic patients were excluded from the study, the positive correlation between BAs and weight was attenuated. However, in obese individuals with prediabetes, there was a significant increase in serum bile acids compared to the metabolically healthy NW group (Figure 1(c)). Additionally, analysis of total BA concentration among only normal-weight participants revealed significantly higher levels in patients in prediabetic condition compared to healthy individuals (univariate *p* = 0.03), albeit group sizes were small. Comparison of normal-weight and obese patients without prediabetes or diabetes showed no significant differences in any of the nine individual BAs (Figure 1(d)). We employed linear regression models to adjust for confounders sex, age and BMI or T2D where applicable (Figure 1(e–h)). In the metabolic health groups, significant associations were observed for female sex and for both the obese (OB) and obese with T2D (OB_T2D) groups, with the normal-weight group serving as the reference (Figure 1(e)). The OB_T2D group exhibited a larger effect size than the OB group without T2D. To separate the influences of weight and T2D, we ran independent models for each predictor, adjusted for sex and age. In the T2D model (Figure 1(f)), female sex and presence of T2D showed significant effects, whereas prediabetes was not independently associated with total BAs after adjusting for sex and age. In the BMI model (Figure 1(g)), female sex was the only significant predictor, indicating that BMI alone does not influence total BAs independently of sex and age. In a model comparing healthy obese subjects to obese subjects with prediabetes (Figure 1(h)), a significant impact was found for the presence of prediabetes but not in the obese group without prediabetes. An additional model with an interaction term for BMI and diabetes status confirmed these results (Supplementary Figure S3). A linear regression model with the covariates BMI, sex and age showed a positive correlation between the HOMA-IR index, a measure of insulin resistance, and high BA concentrations (beta = 0.48, *p* = 2.3e^−3^). In summary, elevated bile acid concentrations are closely linked to diabetes and insulin resistance, suggesting that metabolic and sex-specific factors, rather than BMI alone, play critical roles in influencing BA levels.

The statistical evaluation was conducted using a Kruskal-Wallis test. * *p* < .05, ** *p* < .01, *** *p* < .001, n.s. = not significant

Abbreviations: Akaike Information Criterion (AIC), cholic acid (CA), chenodeoxycholic acid (CDCA), deoxycholic acid (DCA),glycocholate (GCA), glycochenodeoxycholate (GCDCA), glycodeoxycholate (GDCA),taurocholate (TCA), taurochenodeoxycholate (TCDCA), taurodeoxy- cholate (TDCA), underweight (UW), normal weight (NW), obese with type 2 diabetes mellitus (OB_T2D), obese without type 2 diabetes mellitus (OB), type 2 diabetes mellitus (T2D).

### Bile acid composition and conjugation patterns in prediabetes, T2D and obesity

**Figure 2.**
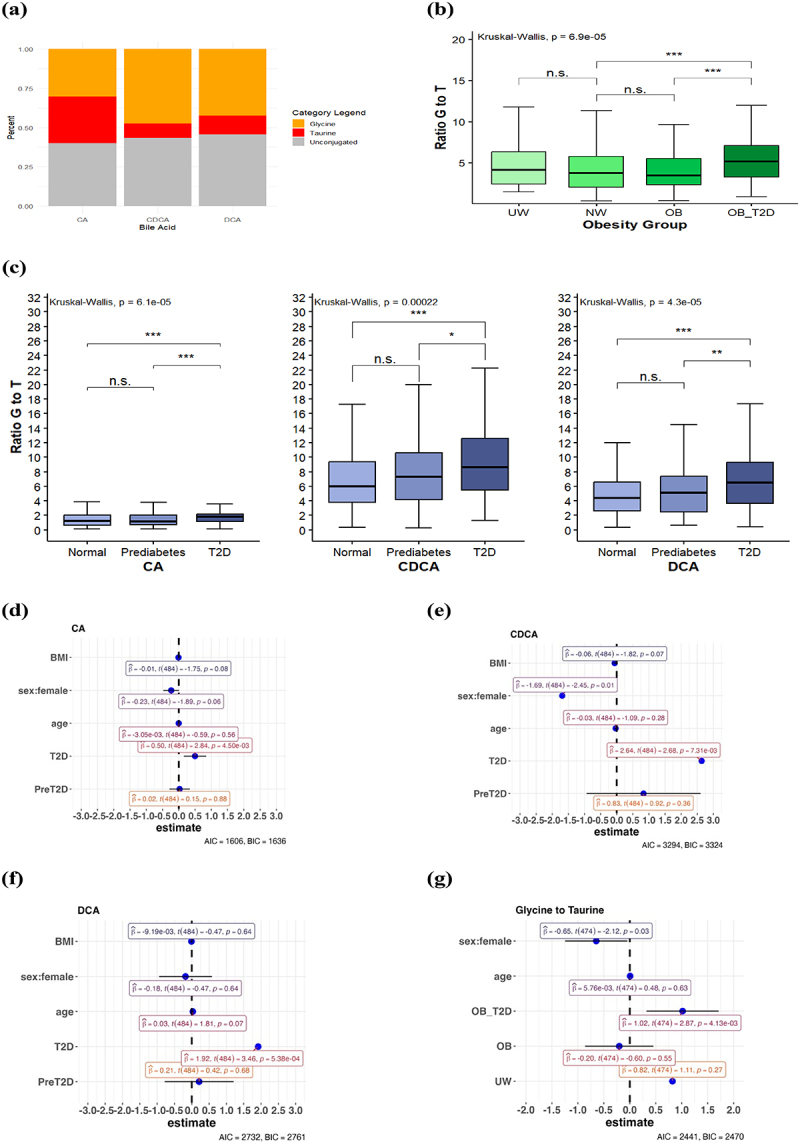
Conjugation of the bile acids (a) Percentages of unconjugated, glycine and taurine- conjugated bile acids in the normal weight group. (b) Ratios of glycine- to taurine- conjugated bile acids in the obesity groups (c) Glycine to taurine ratio of the single bile acids in the diabetes groups. (d-f) Generalized linear regression evaluating the impact of BMI, gender, age and diabetes status for (d) ca/ (e)cdca/ (f) dca on the ratio of glycine to taurine conjugation as the dependent variable. (g) Generalized linear regression evaluating the impact of metabolic status, age and gender on the ratio of glycine to taurine conjugation as the dependent variable.

Having identified that the BA concentration is more related to diabetes state than body weight, we next aimed to examine if conjugation status and the ratio between primary and secondary BAs follow a similar pattern. In our control group, the distribution of unconjugated BAs was 20.89% for CA, 49.19% for CDCA, and 28.02% for DCA. The primary to secondary BA ratio was 73.7%:26.3% in the normal weight group and 72.9%:27.1% in the overall population. A positive correlation was found between T2D and both primary and secondary BAs (*p* = 1.5e^−4^ and 4.3e^−7^, respectively). Primary BAs were elevated in prediabetic and diabetic patients when compared to healthy controls, with no significant differences between prediabetes and diabetes. Secondary BAs did not significantly differ between healthy and prediabetic patients, but were elevated in diabetic patients (*p* = 0.02). The primary-to-secondary BA ratio decreased in T2D groups, suggesting a shift toward more secondary BAs. Obesity had no significant effect on primary or secondary BAs. Regarding BA conjugation, both the normal-weight control group and the overall population showed a 44% unconjugated to 56% conjugated BA ratio. The median ratio of glycine- to taurine-conjugated BAs was 3.77 (IQR = 2.01–5.77) in the normal weight control group and 4.02 (IQR = 2.41–6.13) in the overall population, translating to 78.4% glycine and 21.6% taurine-conjugated bile acids in healthy individuals. Differences in glycine versus taurine conjugation were observed across BA species: in the NW group, CDCA showed the highest glycine conjugation (47%) and lowest taurine conjugation (9%), while CA showed nearly equal glycine and taurine conjugation (30.4% vs. 29.7%) (Figure 2(a)). In patients with T2D and obesity conjugation patterns were similar, with CA consistently exhibiting higher taurine conjugation than CDCA and DCA. A Chi-Square Test confirmed the difference in conjugation between BA metabolites (χ2 = 18.56; *p* = 9.6e^−4^). Diabetic patients had a significant increase in total glycine-conjugated BAs (*p* = 3.9×10^−6^), while total taurine-conjugated BAs did not differ significantly between healthy, prediabetic, and diabetic patients (*p* = 0.75). The glycine-to-taurine conjugation ratio also varied significantly across obesity and diabetes groups, with a relative increase in glycine-conjugated BAs in patients with prediabetes and diabetes. For T2D this trend was reflected in each BA species (CA, CDCA, and DCA) (Figure 2(b,c)). Among patients with obesity, only those with T2D exhibited a higher glycine-to-taurine conjugation ratio. Regression models adjusted for age and sex indicated a pronounced effect of T2D on the glycine-to-taurine ratio, independent of BMI, sex, and age. Figure 2(d–f) present the glycine-to-taurine ratio in CA, CDCA and DCA across obesity groups, adjusted for sex and age, showing a significant effect for the OB_T2D group and female sex, but not for the OB and UW groups. Figure 2(g) analyzes the G:T ratio in diabetes groups adjusted for sex, age, and BMI, with significant independent effects observed for sex and the T2D group. These findings suggest that BA conjugation changes are more strongly associated with prediabetes and T2D than with obesity alone.

The statistical evaluation was conducted using a Kruskal-Wallis test. *p < .05, **p < .01, ***p < .001, n.s. = not significant.

Abbreviations: Akaike Information Criterion (AIC), cholic acid (CA), chenodeoxycholic acid (CDCA), deoxycholic acid (DCA), underweight (UW), normal weight (NW), obese with type 2 diabetes mellitus (OB_T2D), obese without type 2 diabetes mellitus (OB), type 2 diabetes mellitus (T2D).

### Bile acid weighted score (BWS)

Having identified divergent effects of all nine BAs and their conjugation status regarding their interplay with various biomarkers and medical, phenotypic, and lifestyle data, we next decided to additionally apply a “bile acid weighted score” subsuming the effects of the BAs. Supplementary table S4–2 presents correlations between BAs and T2D. The secondary BA DCA had the highest impact on T2D, whereas primary BA GCA was negatively associated with T2D. This aligns with the observed decreased ratio of primary to secondary BAs in diabetic subjects, with significant increases in this ratio from the control group to the prediabetic and T2D groups. The score was calculated using a two-step method: first, an univariate Kruskal-Wallis test to determine which BAs were distinct between the diabetes groups (Table S1:C). Four of the nine BAs showed significant associations: DCA (*p* = 2 × 10 ^*−*6^), GCA (*p* = 1.3×10^*−*5^), GCDCA (*p* = 1.6×10^*−*4^), and GDCA (*p* = 6.8×10^*−*5^). Consequently, we incorporated the estimates of these BAs into the BWS by running ordinal logistic regression models, where estimates were calculated for each BA. Given our previous finding that the variance in BA metabolism can be explained by pre/diabetes rather than obesity, we used diabetes status as the outcome. Secondly, the estimates were subsequently used as a factor for the respective BA, ensuring that all BAs were weighted according to their importance and the direction of their effect. No significant differences were found between prediabetic, and diabetic patients based on the BWS. We then proceeded to analyze the phenotypical data in relation to our BWS, total BAs, and single BAs.

### Bile acid metabolism, anthropometry, sex effects and clinical biochemistry

**Figure 3.**
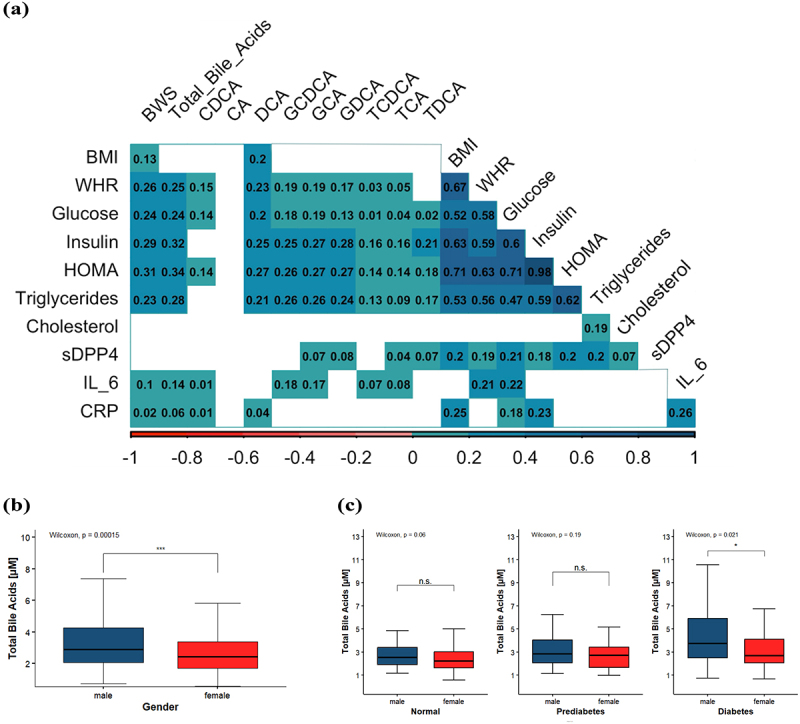
Association of bile acid traits with biological sex and metabolic biomarkers (a) Correlation matrix of bile acid weighted score (BWS), total bile acids and single bile acids with metabolic serum biomarkers. Only significant correlations (fdr >0.05) are displayed. Numbers in boxes refer to correlation coefficients, with darker coloring indicating stronger correlation. List of fdr-corrected p-values can be found in supplement S4–1. (b) total BA levels in males and females (c) Total BA levels in normal, prediabetic and diabetic probands by sex.

In order to gain insights into possible mechanisms on how BAs are related to the diabetes status independent of the body weight we performed additional analyses including biochemical parameters related to glucose metabolism and appetite regulation (Figure 3(a) & S4–1). BMI and WHR: Linear regression revealed a positive correlation between WHR and total BAs (beta = 1.2, *p* = 2.5e^−6^). All individual BAs, except for CA and TDCA, were also correlated with WHR as demonstrated in Figure 3(a). Physical activity and sleep: Activity questionnaire analysis indicated that only CA was correlated with higher daily physical activity, while no other BA was influenced by exercise. Additionally, the BWS was significantly associated with altered sleeping patterns. Insulin Sensitivity: As depicted in Figure 3(a), glucose, insulin, and the HOMA-Index were positively associated with the BWS and all single BAs, except CA (and CDCA for insulin). In a linear regression model, total BAs were positively correlated with insulin (beta = 0.6, *p* = 7e^−4^), with GCA, GCDCA, DCA, GDCA, and TDCA showing significant associations. After adjusting for diabetes status, only GCA and GDCA remained independently associated with insulin (*p* = 0.04 and *p* = 2.0e^−3^). Total BAs and all individual BAs except CA were also positively correlated with serum glucose. Insulin and glucose analyses included adjustments for diabetes status and multiple testing. Incretin signaling: The secretion of GLP1 is stimulated by TGR5 activation by BAs. Fasting GLP1 measurements from 209 participants in the FoCus cohort were available for the present analysis. After excluding 12 patients receiving GLP1-agonists and 15 patients receiving DPP4 inhibitors, 182 subjects remained for the analysis (85 NW and 97 OB_T2D). The measurements ranged from 0.69–49.69 ng/l (Median 6.22 ng/l). GLP1 serum levels showed a positive correlation with the HOMA-Index (*p* = 1.3e^−4^) and BMI (*p* = 7.8e^−5^). Of the single BAs, DCA, GCDCA, and GDCA (*p* = 0.04, 0.03, and 0.03, respectively) were positively associated to GLP1 in linear regression analysis. Total and secondary BAs also positively correlated with GLP1 (*p* = 0.04 and 0.02, respectively), though no association was found with primary BAs. Adjustment for diabetes status reduced the strength of these associations. Cholesterol metabolism: Although the metabolism of cholesterol and BAs is closely linked, total serum cholesterol was not associated with any BAs in either the overall population, the normal-weight or obese group. Inflammatory markers: IL-6 correlated with all primary BAs except for CA, whereas CRP showed the strongest association with secondary BA DCA. In linear regression analysis, including both BMI and diabetes status as confounding variables, the positive association between CRP and IL-6 levels remained significant (*p* = 2.3e^−4^ and *p* = 0.03, respectively). Sex differences: total BAs were higher in men (*p* = 1.5e^−4^). However, significant sex differences were only observed in the OB and OB_T2D groups (*p* = 0.02 and *p* = 0.01, respectively), but not in the NW control group. Among individuals with diabetes, men had higher total BA levels than women (*p* = 0.02), with these patterns illustrated in Figures 1(e-g), 2(d,e), 3(b,c).

Correlation was tested using Spearman’s correlation with fdr-correction for multiple testing. The statistical evaluation for b and c was conducted using a Kruskal-Wallis test. *p < .05, **p < .01, ***p < .001, n.s. = not significant

Abbreviations: body-mass-index (BMI), waist to hip ratio (WHR), Homeostasis Model Assessment Index (HOMA-Index), Dipeptidyl peptidase 4 (sDPP4), interleukin-6 (IL_6), C-reactive Protein (CRP), bile acid weighted score (BWS), cholic acid (CA), chenodeoxycholic acid (CDCA), deoxycholic acid (DCA), glycocholate (GCA), glycochenodeoxycholate (GCDCA), glycodeoxycholate (GDCA), taurocholate (TCA), taurochenodeoxycholate (TCDCA), taurodeoxycholate (TDCA).

Taken together, the association with WHR (abdominal fat), insulin resistance and IL6/CRP might indicate a potential role of BA in inducing and/or maintaining the so-called metabolic inflammation often observed in prediabetes and type 2 diabetes patients.

### Bile acid metabolism and cardiovascular co-morbidities

**Figure 4.**
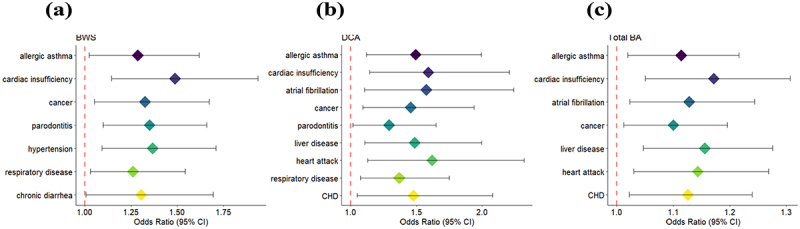
Association of chronic diseases at the baseline (a) bile acid weighted score (BWS) (b) deoxycholic acid (DCA) (c) Total bile acids significance was tested using a logistic regression. Bile acids (BA), coronary heart disease (CHD).

Having identified a potential association of BA with metabolic inflammation we next aimed also to examine cardiovascular disease in relation to BA, since metabolic inflammation is related to this type of chronic lifestyle associated disease as well. Elevated blood concentrations of secondary BAs were significantly associated with common co-morbidities of obesity, prediabetes and T2D (Figure 4). DCA was most strongly associated with heart attack (OR = 1.62, *p* = 0.04) and cardiac insufficiency (OR = 1.59, *p* = 0.04). Other cardiovascular diseases associated with high circulating DC levels included atrial fibrillation, hypertension, and coronary heart disease. Previous studies indicate that secondary BAs may negatively affect cardiac function, with increased ratios of secondary to primary BAs in patients with chronic heart failure.^39^ To explore this, we analyzed primary and secondary BA levels in 27 patients with heart failure, finding significant increases in total and primary BAs (*p* = 4.1e^−3^ and *p* = 3.2e^−3^, respectively), while secondary BA levels and the secondary-to-primary BA ratio were similar between heart failure patients and controls. Given our earlier finding that diabetic patients had higher BA levels than the control group, we also analyzed the diabetes status of our 27 patients with cardiac insufficiency. This sub cohort comprised of three metabolically healthy subjects (one NW), nine patients with prediabetes, and 15 patients with diabetes patients, all of whom were obese. Interestingly, we found that the three non-diabetic subjects had the highest mean BA levels, although no statistical comparison between the groups was feasible because of the small sample size.

### Bile acid metabolism and composition of the gut microbiome

Importantly, the gut microbiome is thought to be a major factor in linking an unhealthy western diet to low-grade metabolic inflammation. Thus, in the next step we aimed to explore the connection between gut microbiome and bile acid composition. We analyzed both inter-and intra-individual variation of the microbiome in relation to circulating bile acid levels. The BA levels were categorized into quartile groups, both in univariate comparisons and when adjusting for confounders. α-diversity, the intra-individual variation of the microbiome, was assessed as species richness in the bile acid quartile groups. Gut microbial species richness differed significantly between the quartile groups of GCDCA (*p* = 0.01) and TDCA (*p* = 0.01). Interestingly, while upper quartile groups with high blood concentrations of TDCA showed increased species richness, high GCDCA serum levels were associated with lower species richness. T2D was also associated with decreased species richness independently as well (*p* = 0.004). Multivariate analysis of species richness and bile acid levels adjusted for T2D revealed that BAs had no independent effect on species richness. β-Diversity was analyzed as the Bray-Curtis dissimilarity index in Principal Coordinate Analysis (PcoA) followed by PERMANOVA. Among the single bile acids quartiles, β-Diversity differed significantly only for secondary BA DCA (Permanova *p* = 0.001). As all secondary bile acids are generated by the microbiome, it is unsurprising that circulating DCA is correlated with changes in microbial composition. BWS, total BAs, and secondary BAs were also related to alterations in β-diversity (PERMANOVA *p* = 0.0002; *p* = 0.0004; *p* = 0.0002). Primary BAs were only nominally significantly associated, thus emphasizing the correlation between secondary BAs and the microbiome. ASVs that differed most between BA quartiles corresponded to the genus *Faecalibacterium* and family *Lachnospiraceae* for the BWS as well as to the genus *Escherichia/Shigella* and to the family *Enterobactericeae* for secondary BA quartiles.

### Host-microbe community metabolism regarding bile acids

Given the important role of the gut microbiome in linking unhealthy dietary habits to metabolic and inflammatory abnormalities in humans, we deepened our analysis by additionally analyzing gut community metabolism. This is important, since gut microbes form complex communities in which species interact in ways ranging from synergy to competition. These interactions involve the exchange of metabolites within the microbial community and between microbes and the host.

**Figure 5.**
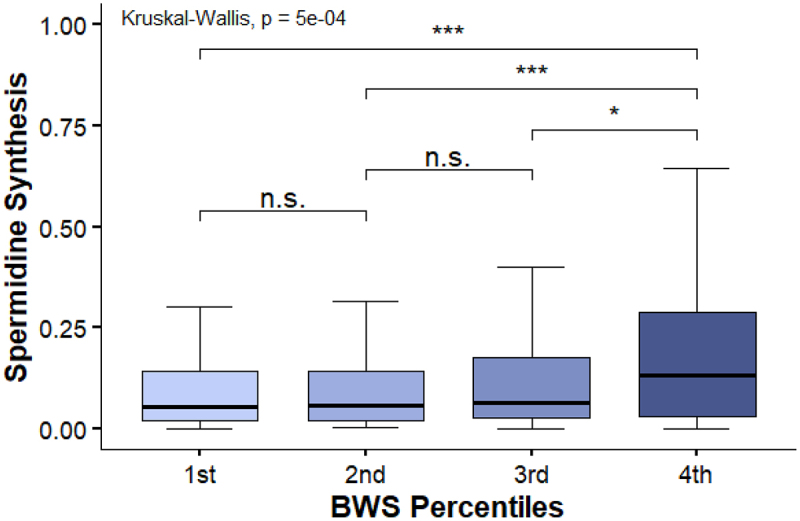
Pathway abundance of Spermidine synthesis in BWS quartiles.

To investigate these dynamics, we employed flux balance analysis (FBA) to model community metabolism, focusing on microbial metabolic pathway abundance, cross-feeding between bacterial species, and bacterial metabolite production. The initial phase of our analysis involved examining differential pathway abundance in the bacterial community across different groups, based on the BWS and individual BAs. We identified 184 nominally differentially abundant pathways related to BWS, 18 of which remained significant after adjusting for multiple testing. The top three pathways in the BWS all belonged to the synthesis of bacterial membranes. 3-deoxy-d-manno-octulosonic acid (KDO) is a sugar that is a substantial part of LPS in the cell membrane of gram-negative bacteria, where LPS is responsible for the protection of the membranes from chemical attacks. A supplementary analysis of pathway abundance in total BA groups additionally showed potential of KDO transfer to lipid IVA in *Haemophilus* sp., in addition to *Escherichia coli* and *Pseudonomas putida*, although all pathways in total BAs were only nominally significant. The top ten differentially abundant pathways for BWS are detailed in Table 1. A noteworthy finding was the significant increase in the microbial spermidine biosynthesis pathway associated with higher BWS, also independent of weight (Figure 5 and linear regression adjusted for confounders: beta = 0.95, p-value = 0.003). Among the single bile acids, only TDCA showed differentially abundant pathways between respective groups. We observed an increase in the microbial L-tyrosine biosynthesis pathway, which was no longer significant after correction for multiple tests.

The statistical evaluation was tested using a Kruskal-Wallis test. *p < .05, **p < .01, ***p < .001, n.s. = not significant.

**Table 1.** Top ten differentially abundant microbial pathways in BWS quartiles, adjusted for BMI, age and sex.

Pathway	Estimate (lin reg)	p-value (FDR)
Kdo transfer to lipid IVA I (E. coli)	0.03	0.004
Kdo transfer to lipid IVA IV (P. putida)	0.03	0.004
UDP-N-acetyl-D-glucosamine biosynthesis I	0.05	6.08e-03
acrylate degradation I	0.02	3.67e-04
L-glutamate degradation II	0.04	0.002
PRPP biosynthesis	0.04	8.23e-05
NADH repair (prokaryotes)	0.01	0.005
urate conversion to allantoin II	0.02	2.8e-04
NADPH repair (prokaryotes)	0.02	0.04
spermidine biosynthesis I	0.09	7.36e-05

Microbial cross-feeding: Linear regression analyses of microbial cross-feeding yielded significant results in relation to the BWS and all single BAs except for CA and GCDCA. The top metabolite in the BWS groups was putrescine, a precursor molecule for spermidine. Putrescine cross-feeding was significantly decreased in subjects with higher BWS (*p* = 0.006). Additionally, cross-feeding of putrescine was significantly decreased in patients with high serum concentrations of CDCA, DCA, and GDCA. Some metabolites were differentially exchanged with respect to a number of single BAs. Two metabolites, galactose and D-arabinose were positively associated with GDCA, TDCA, and TCA. Among the other significantly exchanged bacterial metabolites were eight amino acids: alanine, cysteine, glutamate, lysine, phenylalanine, proline, serine, and tryptophan. There was a significant increase in NADH and NADPH cross-feeding in subjects with high serum GCA concentrations. Bacterial metabolite production: The interplay between the host and the gut microbiome involves the bacterial production of several metabolites, which could potentially be metabolically active in the host. These substances can be predicted *in silico* using FBA. Here, we observed an increase in the production of bacterial metabolites for all BAs, except CDCA. Table 2 shows all metabolites produced in relation to the single BAs, total BAs, and BWS. Of note, the phospholipid L-inositol was associated with both GDCA and TCA.

**Table 2.** Significantly differential production of bacterial metabolites: significance was tested using a linear regression model with fdr correction for multiple testing and adjusted for confounders sex, age and BMI or T2D. Abbreviations: Dihydroxypropane = (S)- 2,3-dihydroxypropane-1-sulfonate, bile acid (BA), cholic acid (CA), chenodeoxycholic acid (CDCA), deoxycholic acid (DCA), glycocholate (GCA), glycochenodeoxycholate (GCDCA), glycodeoxycholate (GDCA),t aurocholate (TCA), taurochenodeoxycholate (TCDCA), taurodeoxycholate (TDCA).

Metabolite	Estimate	adj. p-value (fdr)	BA
Propanal	0.38	2.7e-03	GCA
Hexanoate	0.28	7.4e-04	GCA
L-Inositol	0.14	6.4e-03	GDCA
L-Inositol	0.45	0.003	TCA
Dihydroxypropane	0.25	1.5e-03	TCA
Propanal	0.82	0.006	TCDCA
Hexanoate	0.10	1.3e-03	TCDCA
Dihydroxypropane	0.59	6.9e-03	TDCA
Acetate	0.01	2.8e-04	BWS
Propanal	0.26	0.0001	Total BA
Acetate	0.82	1.6e-03	Total BA
Dihydroxypropane	0.31	2.4 e −02	Total BA

**Figure 6.**
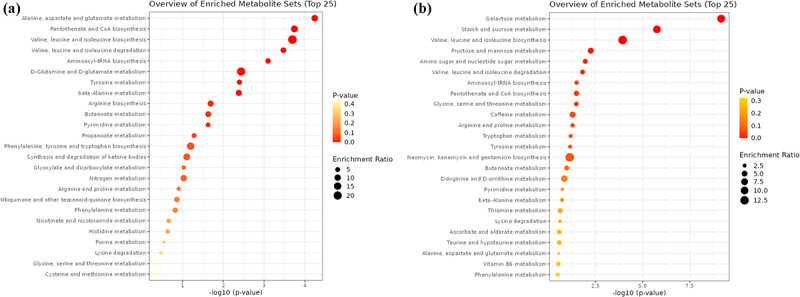
Enriched kegg-pathways a) in the serum and b) in the urine of patients with high circulating total bile acids.

Next, examination of the reciprocal exchange of metabolites between the microbiome and the host prompted an investigation into the potential *in vivo* detection of these predicted metabolites through serum or urine metabolome analysis in human subjects. While our untargeted metabolome analyses and annotations did not encompass all of the metabolites predicted *in silico* and did not result in any significant association between predicted metabolites and actual serum and urine metabolomics, KEGG pathway enrichment analysis revealed the overrepresentation of pathways related to the metabolism of multiple amino acids (serum) and carbohydrate metabolism (urine) (Figure 6).

### Bile acid concentration during surgical and non-surgical weight-loss intervention

**Figure 7.**
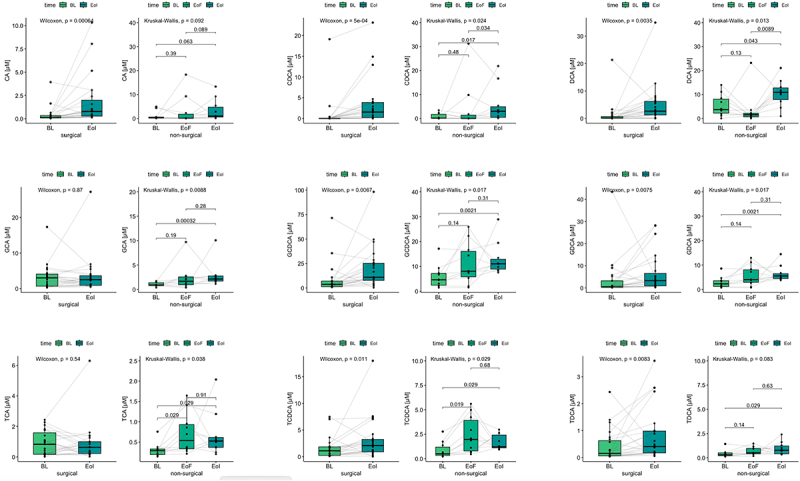
Total bile acid concentration in intervention cohorts. Longitudinal changes in nine BAs during surgical and non-surgical intervention. Associations were tested using paired Wilcoxon and Kruskal-Wallis tests with fdr correction.

Abbreviations: Baseline (BL), End of Fasting (EoF), End of Intervention (EoI); cholic acid (CA), chenodeoxycholic acid (CDCA), deoxycholic acid (DCA), glycocholate (GCA), glycochenodeoxycholate (GCDCA), glycodeoxycholate (GDCA), taurocholate (TCA), taurochenodeoxycholate (TCDCA), taurodeoxycholate (TDCA)

Finally, having found no association of BA with obesity in a steady-state of the body weight, we aimed to examine, if a profound weight loss of obese individuals alters BA serum concentrations. Surgical and non-surgical weight-loss intervention led to a significant increase in each of the nine circulating BAs, with the exception of GCA during surgical intervention which did not differ significantly over time. During non-surgical intervention most BAs increase linearly, while GCA, TCA and DCA were most affected by the fasting phase (Figure 7).

## Discussion

The present study revealed a discernible impact of pre/diabetes status on the BA metabolism. Normal weight prediabetic patients, obese prediabetic and obese diabetic patients exhibited higher total BA serum concentrations than their respective control groups. In patients without prediabetes, body weight did not affect circulating BA levels, suggesting that increased total BAs correlate more strongly with impaired glycemic control than with obesity. In our control group serum samples, we found a mean distribution of 20.89% CA, 49.19% CDCA, and 28.02% DCA for unconjugated BAs aligning with other studies that reported similar ratios in healthy control groups.^40–42^ Several independent studies also found no differences in fasting BA levels between obese and normal-weight patients,^14–16^ highlighting that observed BA elevations may be influenced by glycemic control rather than body weight alone. Lee et al. observed no significant difference in total BA levels between obese individuals compared to normal weight individuals, but observed a remarkable increase from low insulin resistance to insulin resistant individuals regarding total BA and HOMA-Index,^43^ supporting our hypothesis that insulin resistance, prediabetes and diabetes instead of increased body weight are the drivers for the increase in total BAs. Several researchers have also postulated an increase in total BA in T2D or insulin resistance.^17,44–47^ However, no differences in fasting BAs of diabetic patients in comparison to healthy controls were found by other research groups.^19,22^ Most of the studies postulating an effect of body weight on BAs only evaluated the T2D status of their patients, but not insulin resistance or prediabetes. In studies covering the effect of obesity on circulating BAs, we frequently observed increases in fasting glucose, insulin, HbA1c, and HOMA-Index in the obesity group.^12,13,44^ Consequently, it is conceivable that the reported outcomes may have been confounded by glycemic control, which underscores the significance of our findings.

The microbiome converts primary BAs to secondary BAs. The distribution of primary to secondary BAs was 73.7%:26.3% in the normal weight group and 72.9%:27.1% in the overall population. Previous studies have suggested similar ratios of 67:33%.^40,41^ Contrary to the patterns observed in healthy populations, studies on patients with T2D have found that both primary and secondary BAs were elevated. A mouse study on T2D proposed a shift toward a higher proportion of secondary BAs in diabetes.^48^

Several previous studies have suggested a relative increase in secondary BAs in T2D patients.^46^ Our cohort exhibited a similar relationship, with positive associations for both primary and secondary BAs in patients with diabetes and a decreased primary-to-secondary ratio. Again, body weight had no significant impact on the increase in primary and secondary BAs in our main discovery cohort. Interestingly, we observed a mostly opposing effect in the surgical and non-surgical intervention cohort, where circulating levels of most BAs increased with weight-loss. Given that post-intervention samples were taken at the 6 months mark, one might hypothesize that a metabolic steady-state had not yet been achieved. Thus, this observation needs to be validated in a larger cohort, with longitudinal sampling for longer amounts of time and particularly also in the context of T2D and prediabetes, which was not possible in this case. As a result of our study, since short-term interventions are often performed by subjects with obesity, significant changes in BA metabolism have to be considered in future studies whereby conclusions should be drawn only from measurements in a steady state.

The mechanism underlying the relative increase in secondary BAs in patients with diabetes has not yet been elucidated. The composition of primary to secondary BAs can be influenced by the synthesis rate of primary BAs, metabolism of primary to secondary BAs by the gut microbiome, and excretion of the respective fractions. The bacterial transformation of BAs is influenced by the gut microbiome and fiber content of food.^49^ We can therefore hypothesize that differential abundances of microbial species, which have long been associated with T2D, leads to an increase in BA-metabolizing microbes and, hence, an increase in secondary BA production. Decreased primary BAs synthesis seems unlikely, as the results of our previous analyses showed a positive association between T2D and primary BAs. BA conjugation in the liver and intestine, primarily with glycine or taurine, reduces toxicity and enhances its solubility. Consistent differences in glycine versus taurine conjugation patterns across single BA species were noted across all subgroups, with a 44% unconjugated to 56% conjugated BA distribution in both the normal-weight control group and the overall population, in line with the findings of Trottier et al.^40^ In all groups, CA had a significantly higher taurine conjugation percentage than CDCA and DCA, a novel observation to our knowledge.

FXR is involved in modulating CRP release from hepatocytes. Synthetic activators of FXR reduce CRP excretion after stimulation with IL-6 in vitro.^50^ Therefore, we expected a negative correlation between circulating BAs and inflammatory markers. However, in our cohort, BAs were positively associated with inflammatory markers independent of BMI or diabetes status. IL-6 was correlated with BWS and all primary BAs except for CA and CRP was positively associated with the BWS, CDCA, and DCA, the most potent activators of TGR5 and FXR. Similar correlations were established in the NW control group. The Natrium- Taurocholate Cotransporter Peptide (NTCP) is repressed under inflammatory conditions.^2^ Therefore, it is conceivable that the consequent reduction in hepatic BA uptake may explain our positive correlation. In addition, this could be the reason for the association of cardiovascular co-morbidities to BA alterations found in our study, since metabolic inflammation is important in the development of atherosclerosis.

DCA, which impacts bacterial integrity and exerts selective pressure,^9,10^ was expected to decrease species richness in patients with elevated BA levels. However, T2D alone explained variances in species richness, independent of BA levels, with diabetic patients showing significant decreases in species richness, consistent with previous studies.^51^ It remains uncertain whether elevated BAs in T2D mediate these microbiome changes or whether T2D-associated microbial shifts lead to BA alterations. The role of BAs as potential mediators in the pathogenesis of T2D is subject of current discussion. We included both, BMI and T2D, as covariates in our analyses of β-diversity, as both metabolic diseases have been previously associated with microbiome alterations in the past.^7,51^ We found that the diabetes phenotype, along with total and secondary BAs, was associated with changes in β-diversity, independent of BMI.

Our findings in microbial community metabolism identified pathways related to membrane homeostasis as the top differentially abundant pathways associated with BWS. Gram-negative bacteria, which rely on lipopolysaccharides (LPS) for membrane stability, are more susceptible to bile salt effects without LPS.^52^ Additionally, increased D-arabinose cross-feeding was observed with elevated GDCA, TDCA, and TCA concentrations, suggesting a link between BAs and LPS synthesis, with D-arabinose serving as a growth factor for certain bacterial strains.^53^ Furthermore, we noted an elevation in microbial spermidine biosynthesis and a simultaneous reduction in putrescine cross-feeding in individuals with elevated BA concentrations. This activation of microbial spermidine synthesis is probably a response to the detergent properties and oxidative stress caused by BAs. Considering that putrescine serves as a precursor molecule for spermidine biosynthesis, the decrease in bacterial cross-feeding of putrescine might be attributed to amplified microbial spermidine synthesis, leading to a decrease in putrescine availability. These findings on polyamide metabolism are of interest in terms of metabolic diseases, and experimental studies in mice have shown that disruption of polyamine metabolism is associated with impaired glucose and lipid homeostasis. In a mouse model of enhanced polyamine catabolism, Pirinen et al. observed elevated hepatic CYP7A1 expression, increased serum BA concentrations, increased fecal BA excretion, and consequently lower serum cholesterol levels, providing a link between polyamines and BA homeostasis.^54^ Moreover, dietary supplementation with spermidine in nonalcoholic fatty liver disease altered the microbiome and hepatic BA composition, with decreases in secondary BAs.^55^ Furthermore, studies in mice suggest that polyamines are involved in proinsulin biosynthesis in pancreatic β-cells, and that decreased polyamines, as can be found with increasing age and obesity, are contributors to T2D pathogenesis.^56,57^ To the best of our knowledge, no study has been performed on the interplay of BAs and polyamines in relation to the microbiome. Thus, our results led us to then novel hypothesis that high BA concentrations result in increased polyamine synthesis, thereby compensating for the altered microbiome in T2D patients. Additionally, the microbial polyamines could exhibit systemic effects and modulate host BA metabolism. This may represent an additional mechanism for the elevation in circulating BAs levels in patients with diabetes. Further research on the interplay between microbiome-derived polyamines and BA homeostasis is required to better understand the underlying regulatory mechanisms.

We also show that the microbial pathway for synthesis of 5-Phospho-α-D-ribose 1-diphosphate (PRPP) is differentially expressed in subjects with T2D. PRPP plays crucial roles in various physiological processes. It serves as a precursor for purine and pyrimidine biosynthesis, the amino acids L-histidine and L-tryptophan, and nicotinamide adenine dinucleotide (NAD) and nicotinamide adenine dinucleotide phosphate (NADP), which are important co-factors for numerous cellular reactions. A hydroxyl group can spontaneously attach at position 6 to both NAD and NADP to form an NAD(P)H hydrate. This ’damaged’ form cannot exhibit its necessary functions as a co-factor and must be dehydrated by NAD(P) H-dehydratase/epimerase. We observed differential expression of these repair mechanisms in our cohort. Whether BAs increase NAD(P)H hydration has not been investigated. In addition to increased repair, we also observed an increase in NAD cross-feeding in patients with high circulating GCA levels. Regarding this finding we would like to refer to a previous clinical study of our group, showing that a targeted microbiome intervention using controlled ileo-colonic release formulation of nicotinic acid significantly improved insulin sensitivity.^58^

The high correlation observed between bile acids and amino acids in both community metabolism and serum metabolome analyses suggests a link between these two metabolic pathways. Notably, our metabolomic approach revealed the activation of branched-chain amino acid (BCAA) (valine, leucine, and isoleucine) metabolism. This activation aligns with previous studies associating elevated plasma BCAA concentrations with T2D risk.^59,60^ Perturbations in BCAA metabolism have been implicated in interfering with insulin and glucose homeostasis, making branched-chain amino acids potential early biomarkers for identifying individuals at risk of developing T2D. Consistent with this, the Framingham Offspring study demonstrated the predictive value of plasma BCAA, along with aromatic amino acids, such as tyrosine and phenylalanine, in anticipating future T2D.^61^ Again, our findings resonate with these established connections, as we observed increased expression of the microbial tyrosine biosynthesis pathway, enhanced bacterial phenylalanine cross-feeding and activation of the KEGG pathway associated with tyrosine metabolism.

### Limitations

Like many studies, our microbiome analysis relies on amplicon sequencing of the V1-V2 region of the 16S gene. This type of high-throughput analysis, while extremely common in the scientific community, is associated with a number of well-documented pitfalls and sources of unwanted technical variation which make comparison of results from different studies challenging. In particular, the choice to sequence only one variable region is a historical cost-efficiency compromise. While this particular region has been proven to provide robust results at genus level data analysis and in biomedical environments, it could be argued that the resolution and interpretability of our results could be improved by choosing another sequencing region such as V3-V4 or V1-V3. Hence, for future analysis we suggest adopting technical guidelines of the International Microbiome Standards (IHMS) to support homogenization of human microbiome studies. Similarly, it must be noted that our analysis of gut microbial functions and community metabolism is purely in silico, based on amplicon sequencing and might thus be biased by the aforementioned sequencing and bioinformatic choices. Additionally, our BA analysis is based on relative abundance values and not all circulating BA species could be detected, which should be considered when comparing with other studies. Finally, for our intervention study no data at the time after reaching a metabolic steady state were available. Thus, further studies are needed to examine if after long term weight loss the different association of glycemic control versus weight become prominent again.

## Conclusion

Our findings indicate that abnormalities in glucose metabolism (insulin resistance, prediabetes, type 2 diabetes) occurring with obesity rather than solely an increase in body weight are related to alterations in BA metabolism, whereby metabolic inflammation and the gut microbiome are potential mediating factors. To the best of our knowledge, we for the first time provide novel insights into the interactions between microbial and host metabolites in relation to human BA metabolism in diabetic subjects, particularly regarding polyamine and amino acid pathways. While many studies claim changes in the gut microbiome in relation to diabetes to be mostly negative, the up-regulation of the microbial spermidine production in relation to the BWS found in our study might suggest also beneficial/compensatory effects of altered microbial communities in diabetic subjects, especially since spermidine has recently been shown to suppress diabetes onset and progression by modulating RIPK1-mediated cell death and inflammation.^62^ From our point of view, this aspect should be addressed in future translational and/or mechanistic studies

## Supplementary Material

Supp_BA_paper_rev2.xlsx

## Data Availability

The complete dataset used in this study is stored at the biobank P2N in agreement with German data privacy laws and can be requested following the procedure described at https://portal.popgen.de.
